# Gastroesophageal Reflux Disease in a One-Week-Old Infant Presenting With Cyanosis and Respiratory Distress

**DOI:** 10.7759/cureus.29632

**Published:** 2022-09-26

**Authors:** Max Ledersnaider, Norma Kreilein, Renee Triplett, Nicholas J Peterman

**Affiliations:** 1 Medicine, Carle Foundation Hospital, Urbana, USA; 2 Pediatrics, Carle Richland Memorial Hospital, Olney, USA; 3 Pediatrics, Ascension St. Vincent Hospital, Evansville, USA

**Keywords:** respiratory distress, infant, perioral cyanosis, gastroesophageal reflux disease, physiologic gastroesophageal reflux

## Abstract

Gastroesophageal reflux (GER) is a common occurrence in infancy and early childhood. While GER is considered physiologic, gastroesophageal reflux disease (GERD) can result when extensive GER leads to troublesome symptoms such as choking, gagging, vomiting, refusal to feed, and poor weight gain. In extreme cases, GERD can cause severe respiratory complications such as apnea and aspiration pneumonia. We present the case of a one-week-old Amish female who had no prenatal care and presented with severe hypoxemia, tachypnea, and costal retractions. Further history from the family revealed persistent irregular breathing, sweating during feeds, and episodic perioral cyanosis. The patient required stabilization in the intensive care unit and received an extensive workup to rule out sepsis, cyanotic heart disease, other infectious etiologies, and other common causes of respiratory distress. The patient underwent a modified barium swallow study and was diagnosed with aspiration pneumonitis resulting from GERD and oropharyngeal dysphagia. Infantile cyanosis and respiratory distress can be manifestations of a variety of underlying illnesses. Once common causes of cyanosis have been excluded, GERD or disordered feeding should be considered as a potential etiology.

## Introduction

Gastroesophageal reflux (GER) is common in infancy and early childhood. While GER is considered physiologic, gastroesophageal reflux disease (GERD) can result when extensive GER leads to troublesome symptoms such as choking, gagging, vomiting, refusal to feed, and poor weight gain. In extreme cases, GERD can cause severe respiratory complications such as apnea and aspiration pneumonia [1]. Here, we present a severe case of GERD in a one-week-old female that presented with cyanosis and respiratory distress, ultimately requiring stabilization in the intensive care unit (ICU) prior to diagnosis. This case is of interest due to both the severity and early presentation of the symptoms.

## Case presentation

A one-week-old Amish female born at 40 weeks and three days gestation via home vaginal birth without any reported complications presented after her mother noticed that the patient was “struggling to breathe.” Maternal/gestational history was unknown as there was no routine prenatal care, and pulse oximetry was not performed at birth or throughout her first week of life. The parents noted that the infant had been breathing “hard and fast” since birth, but they believed that was normal. The parents also reported some episodes of perioral cyanosis, irritability, and sweatiness during feeds. However, they noted that the patient had been breastfed “frequently” and had produced an average of 10 wet diapers per day.

On physical examination, the patient weighed 2.96 kg with an unknown birthweight, afebrile, with an average respiration rate of 79 breaths/minute, an average pulse of 125 beats/minute, and oxygen saturation of 70-80% on room air. Oxygen saturation improved to 95% once 1.5 L of oxygen was delivered via a simple nasal cannula. Auscultation of the lungs revealed reduced breath sounds bilaterally throughout the lung fields with tachypnea and grunting breaths and intercostal retractions. No rales or wheezes were present. The nose and mouth appeared normal on the examination. The patient’s skin was mildly jaundiced with physiologically dry skin consistent with a one-week-old infant, and no perioral, mucous membrane, or extremity cyanosis was present. Cardiovascular, abdominal, and neurological examinations were unremarkable.

Initial lab studies revealed a white blood cell count of 28.5 × 10^9^/L with 4% bands and an absolute neutrophil count of 18.24 × 10^9^/L. Additional lab studies revealed total bilirubin of 13.6 mg/dL, serum potassium of 5.5 mmol/L, and serum HCO^3-^ of 17 mmol/L. Venous blood gas was drawn which revealed a pH of 7.54, pCO_2_ of 24 mmHg, and pO_2_ of 212 mmHg. Chest X-ray showed hyperinflated lungs and flattened diaphragm with a normal cardiac silhouette. Blood cultures and respiratory pathogen detection panels were sent. The patient received one dose of empiric ampicillin and gentamicin before being transferred to a pediatric ICU for further evaluation and management.

The differential diagnosis for the patient’s presentation included sepsis, infectious pneumonia (viral vs. bacterial), congenital cyanotic heart disease, transient tachypnea of the newborn, neonatal respiratory distress syndrome, aspiration pneumonitis, esophageal atresia, tracheoesophageal fistula, and inborn error of metabolism such as galactosemia.

**Figure 1:**
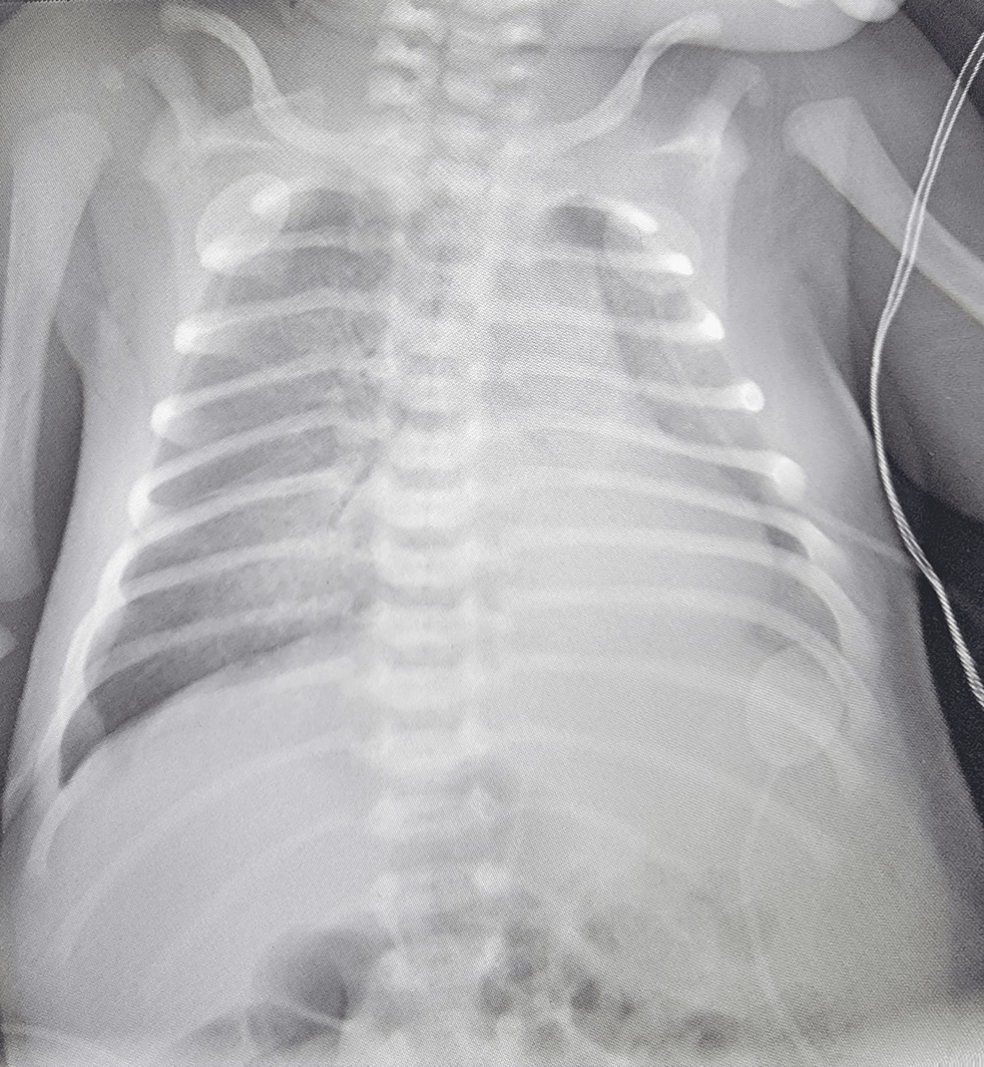
Chest X-ray.

A repeat chest X-ray was performed four hours later upon arrival to the ICU which showed diffuse airspace disease most concerning for pulmonary edema or bilateral pneumonia (Figure 1). These findings together with the reported history of cyanosis with feeding, persistent hypoxia, and abnormal respiration were cause for concern of cyanotic heart disease. An echocardiogram and electrocardiogram were completed but revealed no evidence of congenital cyanotic heart disease in the patient.

Given the leukocytosis on presentation, there was a high concern for infection. Additional blood and urine samples were obtained and sent for culture. As the patient was found to have an elevated liver aspartate transaminase (AST) of 70 U/L (normal 0-37 U/L), herpes simplex virus (HSV)-1 and HSV-2 polymerase chain reaction (PCR) were sent from a blood sample as well as corneal, genital, and nasal swabs. Antibiotic therapy was transitioned to ampicillin and ceftazidime for broader coverage. Acyclovir was added for empiric HSV coverage while PCR results were pending. Acyclovir was discontinued when all viral PCR samples returned negative.

Despite the absence of fever on presentation, infectious meningitis in the setting of neonatal sepsis was considered. A head ultrasound was performed and revealed no abnormalities. Lumbar puncture was not performed given lesser concern for infectious meningitis following an unremarkable head ultrasound.

The patient’s elevated AST, in combination with her demographic (Amish), and lack of prenatal care, there was a raised suspicion of metabolic disorder. On further evaluation, ammonia and lactic acid were found to be normal, and there was no reported family history of metabolic or dietary conditions.

Aspiration was also considered as a potential etiology of the patient’s leukocytosis and abnormal chest X-ray. To assess any functional swallowing difficulties, the patient was evaluated by the speech-language pathology team. She was found to have oropharyngeal dysphagia with discoordination during swallowing that was consistent with infantile reflux regurgitation. A modified barium swallow test was performed but was normal and did not reveal any frank aspiration.

The patient received a diagnosis of aspiration pneumonitis versus aspiration pneumonia secondary to disordered feeding and GERD of the newborn. The patient’s acute hypoxia was treated with 1 L oxygen via a nasal cannula. Subsequently, a 10 mL/kg intravenous (IV) fluid bolus of normal saline was given and maintenance IV fluids were initiated while the patient was held on a nothing by mouth (NPO) order. Supplemental oxygen and IV fluids were discontinued by the time of discharge.

After five days of incubation, no pathogen growth was noted in the blood or urine samples. An additional respiratory pathogen panel (including respiratory syncytial virus, influenza A/B, and coronavirus disease 2019) also returned negative. The patient completed six days of ampicillin and ceftazidime and one day of acyclovir by the time of discharge.

To address the patient’s reflux during feeds, she was transitioned from breastmilk to thickened infant formula which was well tolerated. The patient’s family was interested in continuing with breastfeeds after discharge. They were instructed to thicken the feeds for the patient by mixing oatmeal into expressed breastmilk as they were limited in other thickening agents in the Amish community.

At the time of discharge, the patient appeared well and in no acute distress. The tachypnea and retracted breathing had resolved, and pulmonary auscultation revealed no rales or wheezes. The patient’s leukocytosis had also resolved, and she continued to be afebrile. There was a discussion of home medications for the patient’s reflux, but the parents believed the thickened feeds would be sufficient. Overall, the patient had gained 295 g during her admission and was discharged at a weight of 3,255 g.

## Discussion

Physiologic GER with feeding, sometimes referred to as reflux regurgitation, is a common problem for neonates and infants. Prevalence of regurgitation affects as many as 86.7% of infants less than one-year-old with peak incidence at two months of age. Physiologic GER usually resolves by one year of age without medical intervention and long-term sequelae [2].

On the other hand, the exact incidence of GERD is difficult to discern given the wide variation in severity and the frequency of troublesome symptoms that affect the quality of life or cause pathologic complications [1]. Not all infants exhibit symptoms recognizable to caregivers or medical providers, such as coughing or choking, when they regurgitate [3]. Reports indicate that GERD prevalence may range from 2% to 26% of infants, with a peak incidence around one month of age [1,3-5]. Complications of GERD vary from relatively mild disturbances, such as crying, irritability, or emesis, to potentially life-threatening symptoms, such as apnea, failure to thrive, aspiration syndromes, and recurrent pneumonia [1].

Cyanosis in newborns is relatively common among infants and is generally categorized as either peripheral or central. Peripheral cyanosis, sometimes called acrocyanosis, is often physiologic in nature due to large arteriovenous gradients or slow flow through capillary beds. Central cyanosis, however, usually indicates a serious or life-threatening process and requires urgent evaluation of potential cardiac, infectious, pulmonary, nervous, hematologic, anatomic, or metabolic causes [6]. Similarly, respiratory distress can be seen in a variety of etiologies, including pulmonary, cardiac, infectious, metabolic, and systemic diseases. Timely intervention to support the child’s breathing and diagnosis of the underlying condition is critical to prevent complications, such as chronic lung disease, respiratory failure, and even death [7]. Once other common etiologies have been excluded, GERD or disordered feeding syndromes, as opposed to physiologic GER, should be considered as a cause of cyanosis and respiratory distress in the infant [8].

Several studies have found strong links between the prevalence of airway disease, including aspiration syndromes, and GERD, with some studies suggesting multiple pathophysiological mechanisms to explain causality [9-11]. Lack of recognition or failure to treat aspiration syndrome is a large potential source of morbidity among infants with GERD. The most common complication of aspiration syndrome is chemical pneumonitis, but other sequelae can include atelectasis, lung abscess, pneumonia, empyema, pneumothorax, malnutrition, septicemia, and shock [12]. In extreme cases, infants may require prolonged mechanical ventilation and hospitalization [12]. Importantly, one study comparing children with community-acquired pneumonia to children with aspiration pneumonia found that aspiration pneumonia accounts for longer hospital stays, increased ICU admission, and correlates with medically complex cases [11].

There is no definitive list of risk factors or predisposing conditions of aspiration syndrome though the most common include accidental ingestion, altered consciousness, anatomic malformations, and neurological disorders [12]. Children who aspirate oropharyngeal contents also carry greater odds of eventually requiring mechanical ventilation than children who aspirate other fluids or particulate matter [13].

The diagnostic approach for GERD-related aspiration syndrome in infants should include clinical observation of a feeding session as well as a video-fluoroscopic swallow study, modified barium swallow, or fiberoptic endoscopic evaluation of swallowing [14]. The role of imaging such as chest X-ray or computed tomography in evaluating dysfunctional swallowing is limited but can be useful in identifying lung disease [14].

## Conclusions

GERD is distinguished from physiologic GER by the presence of troublesome symptoms that affect the quality of life or cause pathologic complications. Without timely identification and treatment of aspiration, these infants are subject to increased risk of developing aspiration syndrome, pneumonia, and respiratory failure. When an infant presents with cyanosis and respiratory distress, GERD is an important potential etiology that should be considered when cardiac, respiratory, metabolic, and other etiologies have been excluded.

## References

[1] Rybak A, Pesce M, Thapar N, Borrelli O (2017). Gastro-esophageal reflux in children. Int J Mol Sci.

[2] Osatakul S, Sriplung H, Puetpaiboon A, Junjana CO, Chamnongpakdi S (2002). Prevalence and natural course of gastroesophageal reflux symptoms: a 1-year cohort study in Thai infants. J Pediatr Gastroenterol Nutr.

[3] Nelson SP, Chen EH, Syniar GM, Christoffel KK (1997). Prevalence of symptoms of gastroesophageal reflux during infancy. A pediatric practice-based survey. Pediatric Practice Research Group. Arch Pediatr Adolesc Med.

[4] Van Howe RS, Storms MR (2010). Gastroesophageal reflux symptoms in infants in a rural population: longitudinal data over the first six months. BMC Pediatr.

[5] Gulati IK, Jadcherla SR (2019). Gastroesophageal reflux disease in the neonatal intensive care unit infant: who needs to be treated and what approach is beneficial?. Pediatr Clin North Am.

[6] Steinhorn RH (2008). Evaluation and management of the cyanotic neonate. Clin Pediatr Emerg Med.

[7] Reuter S, Moser C, Baack M (2014). Respiratory distress in the newborn. Pediatr Rev.

[8] Epifanio M, Eloi J, Cassiano AS, Pinheiro D, Spolidoro JV (2014). Infants under 3 months old with cyanosis at the emergency room: could it be gastroesophageal reflux?. Dis Esophagus.

[9] Jadcherla SR (2006). Upstream effect of esophageal distention: effect on airway. Curr Gastroenterol Rep.

[10] del Rosario JF, Orenstein SR (1996). Evaluation and management of gastroesophageal reflux and pulmonary disease. Curr Opin Pediatr.

[11] Hirsch AW, Monuteaux MC, Fruchtman G, Bachur RG, Neuman MI (2016). Characteristics of children hospitalized with aspiration pneumonia. Hosp Pediatr.

[12] Karim RM, Momin IA, Lalani II, Merchant SS, Sewani AA, Hassan BS, Mahmood N (1999). Aspiration pneumonia in pediatric age group: etiology, predisposing factors and clinical outcome. J Pak Med Assoc.

[13] Bowman OJ, Hagan JL, Toruno RM, Wiggin MM (2020). Identifying aspiration among infants in neonatal intensive care units through occupational therapy feeding evaluations. Am J Occup Ther.

[14] Tutor JD, Gosa MM (2012). Dysphagia and aspiration in children. Pediatr Pulmonol.

